# Late-stage Anle138b treatment ameliorates tau pathology and metabolic decline in a mouse model of human Alzheimer’s disease tau

**DOI:** 10.1186/s13195-019-0522-z

**Published:** 2019-08-01

**Authors:** Matthias Brendel, Maximilian Deussing, Tanja Blume, Lena Kaiser, Federico Probst, Felix Overhoff, Finn Peters, Barbara von Ungern-Sternberg, Sergey Ryazanov, Andrei Leonov, Christian Griesinger, Andreas Zwergal, Johannes Levin, Peter Bartenstein, Igor Yakushev, Paul Cumming, Guido Boening, Sibylle Ziegler, Jochen Herms, Armin Giese, Axel Rominger

**Affiliations:** 1 Department of Nuclear Medicine, University Hospital, LMU Munich, Marchioninistr.15, 81377 Munich, Germany; 2 German Center for Neurodegenerative Diseases (DZNE), Munich, Germany; 3 Max Planck Institute for Biophysical Chemistry, Göttingen, Germany; 4 Department of Neurology, University Hospital, LMU Munich, Munich, Germany; 5 Munich Cluster for Systems Neurology (SyNergy), Munich, Germany; 6 Neuroimaging Center (TUM-NIC), Technische Universität München, Munich, Germany; 7 Department of Nuclear Medicine, Technical University of Munich, Munich, Germany; 8 Department of Nuclear Medicine, Inselspital Bern, Bern, Switzerland; 9 School of Psychology and Counselling and IHBI, Queensland University of Technology, Brisbane, Australia; 10 Center for Neuropathology and Prion Research, Ludwig-Maximilians-Universität, Feodor Lynen-Str. 23, 81377 Munich, Germany; 11 MODAG GmbH, 55324 Wendelsheim, Germany; 12 DFG Research Centre Nanoscale Microscopy and Molecular Physiology of the Brain, 37070 Göttingen, Germany; 13 Cluster of Excellence “Multiscale Bioimaging: from Molecular Machines to Networks of Excitable Cells” (MBExC), University of Göttingen, Göttingen, Germany

**Keywords:** Tau, Neuronal injury, Late-stage, Anle138b, Small animal PET

## Abstract

**Background:**

Augmenting the brain clearance of toxic oligomers with small molecule modulators constitutes a promising therapeutic concept against tau deposition. However, there has been no test of this concept in animal models of Alzheimer’s disease (AD) with initiation at a late disease stage. Thus, we aimed to investigate the effects of interventional late-stage Anle138b treatment, which previously indicated great potential to inhibit oligomer accumulation by binding of pathological aggregates, on the metabolic decline in transgenic mice with established tauopathy in a longitudinal ^18^F-fluorodeoxyglucose positron emission tomography (FDG-PET) study.

**Methods:**

Twelve transgenic mice expressing all six human tau isoforms (hTau) and ten controls were imaged by FDG-PET at baseline (14.5 months), followed by randomization into Anle138b treatment and vehicle groups for 3 months. FDG-PET was repeated after treatment for 3 months, and brains were analyzed by tau immunohistochemistry. Longitudinal changes of glucose metabolism were compared between study groups, and the end point tau load was correlated with individual FDG-PET findings.

**Results:**

Tau pathology was significantly ameliorated by late-stage Anle138b treatment when compared to vehicle (frontal cortex − 53%, *p* < 0.001; hippocampus − 59%, *p* < 0.005). FDG-PET revealed a reversal of metabolic decline during Anle138b treatment, whereas the vehicle group showed ongoing deterioration. End point glucose metabolism in the brain of hTau mice had a strong correlation with tau deposition measured by immunohistochemistry (*R* = 0.92, *p* < 0.001).

**Conclusion:**

Late-stage oligomer modulation effectively ameliorated tau pathology in hTau mice and rescued metabolic function. Molecular imaging by FDG-PET can serve for monitoring effects of Anle138b treatment.

**Electronic supplementary material:**

The online version of this article (10.1186/s13195-019-0522-z) contains supplementary material, which is available to authorized users.

## Background

Neurofibrillary tangles constitute one of the most characteristic neuropathological findings in Alzheimer’s disease (AD), which is the most frequent form of dementia, and tangles likewise occur in certain non-AD dementias known collectively as tauopathies [1]. Several in vivo investigations have confirmed a strong link between the presence of hyperphosphorylated tau in the brain, which self-aggregates to paired helical tau filaments and neurofibrillary tangles [2], and future metabolic and cognitive declines in preclinical AD [3–5]. Thus, there is great impetus to develop effective interventions against tau deposition in AD and other neurodegenerative diseases.

Preventive treatment strategies with small molecular weight oligomer modulators of tau, which comprise a suitable pharmacokinetic profile and possess good brain penetrance [6], present one promising pharmacological approach to ameliorate tau pathology. This strategy has shown early success in inhibiting tau aggregation in vitro and in reducing 4R tau aggregates in P301S mice, which serve as a model for tau-positive frontotemporal dementia [7]. The same treatment also attenuated intracerebral accumulations of α-synuclein in a mouse model of Parkinson’s disease [8] and β-amyloid in AD model mice [9]. However, there has hitherto been no direct confirmation of beneficial impacts of oligomer modulator treatment on brain function and tau protein accumulation in transgenic mice expressing human tau protein. Furthermore, the efficacy of late-stage oligomer modulation which emulates a more relevant clinical setting is also unproven.

Molecular brain imaging with ^18^F-fluorodeoxyglucose positron emission tomography (FDG-PET) is a powerful in vivo tool for revealing metabolic deficits in neurodegenerative diseases and has now attained acceptance in the clinical diagnosis of AD [10–12]. Longitudinal FDG-PET is also fit for the preclinical assessment of neuronal injury in a mouse model of tauopathy [13]. Given this background, we aimed to perform an interventional treatment study with the novel aggregation-inhibiting oligomer modulator Anle138b in transgenic mice expressing all six human tau isoforms (hTau), thus constituting a murine model of AD tauopathy [14]. We initiated the treatment at a late disease stage characterized by the presence of tau aggregates, thus emulating a likely clinical scenario. Our main end point for testing the effectiveness of the treatment was serial monitoring of cerebral metabolism by FDG-PET. In addition, we made an end point immunohistochemical assessment of the density of tau pathology after 3 months’ treatment with Anle138b as compared to untreated hTau mice. Finally, we undertook a correlational analysis of the relationship between individual tau pathology with longitudinal metabolic decline to FDG-PET.

## Materials and methods

### Animals and study design

All experiments were carried out in compliance with the German national guidelines for animal protection (TierSchG, Germany) and with approval of the local animal care committee (Regierung von Oberbayern), under supervision by a veterinarian. Experiments, analyses, and reporting were performed in accordance with the ARRIVE guidelines [15]. Animals were housed in a temperature- and humidity-controlled environment with a 12-h light–dark cycle, with free access to food (Ssniff, Soest, Germany) and water. Sample size calculation (G*Power, V3.1.9.2, University of Kiel, Germany) was based on earlier FDG-PET estimates in mice and assuming a type I error *α* = 0.05, a power of 0.8, and a dropout rate of 15% during follow-up. Assuming a relevant treatment effect of 5%, we calculated a group size of six, including one mouse for dropout compensation.

Twelve hTau mice and ten controls (all female) were purchased from Jackson Laboratories (JAX®) at 3 months of age and housed in our pathogen-free animal facility until attaining 14.5 months of age. We then undertook baseline FDG-PET imaging and randomized the mice into Anle138b treatment and vehicle treatment groups (*n* = 6/5 each). hTau mice had been designed to express all six isoforms of human tau in similar ratios to that in the diseased human brain, but do not express significant amount of mouse tau [14]. The model was generated by crossing 8c mice that express a tau transgene derived from a human P1-derived artificial chromosome, H1 haplotype [16] together with tau knock-out mice that have a targeted disruption of exon one of the tau gene [17].

The hTau mice that we used are homozygous for Mapt <tm1(EGFP)Klt> and heterozygous for Tg (NAPT)8cPdav, whereas controls only contain the homozygous Mapt <tm1(EGFP)Klt> mutation. Anle138b treatment was administered for 3 months, and follow-up PET imaging was performed in the last week of treatment. After a visual quality check of PET images, we performed transcardial perfusion with fixation in 4% paraformaldehyde and brain extraction for immunohistochemical analyses.

### Anle138b treatment

We used the oligomer modulator Anle138b ([3-(1,3-benzodioxol-5-yl)-5-(3-bromophenyl)-1*H*-pyrazole]) [6] as a therapeutic against tau deposition. The compound was formulated in food pellets (Ssniff, Soest, Germany) at a concentration of 2 g/kg pellets [7], which were administered ad libitum over a period of 3 months. The food composition was based on regular maintenance diet (16.2 MJ/kg; 9% fat, 24% protein, 67% carbohydrates). Detailed specifications of the Anle138b formulation can be found in supplement #2 of [6]. In brief, (1-(1,3-benzodioxol-5-yl)-3-(3-bromophenyl)propane-1,3-dione), a previously reported pyrazole title compound [18], was used as a precursor for the chemical synthesis of Anle138b in several steps. Control mice were fed with unmodified food pellets.

### PET imaging

#### PET data acquisition and analyses

All PET procedures followed standardized, established protocols [19]. In brief, mice were anesthetized with isoflurane (1.5%, delivered at 3.5 l/min) and placed in the aperture of the Siemens Inveon DPET [20] as described previously [21]. Mice had been fasted for at least 3 hours prior to tracer administration. Static FDG-PET imaging from 30 to 60 min p.i. was performed after administration of 13.2 ± 2.1 MBq ^18^F-FDG as previously established [19]. The emission recording was followed by a 15-min transmission scan using rotating ^57^Co point sources. The image was reconstructed as a single 30-min frame in 4 OSEM3D and 32 MAP 3D iterations, giving a target resolution of 1.0 mm and a zoom factor of 1.0, with scatter-, attenuation-, and decay-correction, resulting in a final voxel dimension of 0.78 × 0.78 × 0.80 mm. Following recovery from anesthesia, mice were returned to their home cages.

#### PET post-processing

FDG-PET images (30–60 min) were co-registered to an MRI mouse brain atlas [22] by a manual rigid-body transformation (TX_rigid_) using the PMOD fusion tool (V3.5, PMOD Technologies Ltd.). A reader who was blind to the type of mouse confirmed the initial registration. Then, we applied a reader-independent co-registration by generating treatment group-specific standard FDG-PET templates in the MRI atlas space. Non-linear brain normalization was performed with the PMOD fusion tool for each individual co-registered image to obtain a transformation matrix (TX_BrainNorm_) for each mouse brain to the template. The manual (TX_rigid_) and automatic (TX_BrainNorm_) transformations were concatenated and applied to the native space μPET data to guarantee a minimum of interpolation. As the μPET templates had been initially aligned to the atlas, all final fused μPET images had the same spatial orientation and voxel dimensions as the MRI mouse brain atlas, i.e., 0.064 × 0.064 × 0.064 mm.

#### PET analysis

An oval-shaped frontal cortical volume of interest (VOI; 28 mm^3^) and a bilateral circular-shaped hippocampal VOI (11 mm^3^) defined in the MRI atlas were placed on the resampled image to calculate the mean radioactivity concentrations in standardized uptake value (SUV) units. Absence of tau pathology in the brainstem of hTau mice has been shown earlier [14], justifying its use as a reference tissue in this mouse model. We calculated the SUV ratios (SUVR_BST_) as the ratio of the radioactivity concentrations in target and brainstem reference VOIs of the Mirrione atlas implemented in PMOD [23]. Longitudinal changes were computed for each mouse as the absolute difference of SUVR values between baseline and follow-up scans (ΔSUVR).

### Immunohistochemical analyses

After removal from the skull, brains were bisected at the midline and one cerebral hemisphere randomly selected for immunohistochemical analysis after fixation by immersion in 4% paraformaldehyde at 4 °C for 24 h. A mean of three representative 50-μm-thick slices per animal was then cut in the sagittal plane between 1.5 and 2.0 mm from the midline using a vibratome (VT 1000 S, Leica, Wetzlar, Germany). Free-floating sections were permeabilized with 2% Triton X-100 overnight and then blocked with I-Block™ Protein-Based Blocking Reagent (Thermo Fischer Scientific). We obtained immunohistochemical labelling of hTau using the CP13 primary antibody (dilution; 1:25, 48 h, RT), which binds specifically to phosphorylated serine 202 (pS202) on tau, followed by incubation with the A-21244 secondary antibody, which contains Alexa Fluor 647 dye (Invitrogen, 1:500) [16, 24]. The unbound dye was removed by three washing steps with PBS, and the slices were then mounted on microscope slides with fluorescent mounting medium (Dako, Germany). Images were acquired with an inverted confocal Laser-Scanning Microscope LSM510=NLO (Zeiss). We imaged the frontal cortex and hippocampus of each slice three-dimensionally in tile scan mode, which allows automatic stitching of an array of fields of view. Tau load (%) in maximum intensity projected image stacks was calculated as the summed area of all tau-positive cells identified using an automated intensity threshold relative to the total inspected area in ImageJ software (Wayne Rasband, (NIH)). Second, we calculated the number of CP13-positive neurons per area (N/mm^2^) in the cortex and the hippocampus. The operator was blind to the PET results.

### Statistics

Statistics were performed using SPSS (V25, IBM Cooperation) and GraphPad Prism (V5.03). FDG-PET measures of baseline, follow-up assessment, and longitudinal changes, as well as tau burden (%), the number of tau-positive neurons per area and body weight, were compared between the three groups of treated hTau mice, vehicle hTau mice, and non-carrier controls by a one-way ANOVA including Tukey post hoc correction for multiple comparisons. Longitudinal FDG-PET measures in the treatment group were also tested voxel-wise (paired *t* test) by a statistical parametric mapping approach [25] to allow a region-independent evaluation of metabolic rescue. Body weight was compared by an unpaired Student’s *t* test. For inter-modality correlation analyses, Pearson coefficients of correlation (*R*) were calculated between region-specific tau area (%) values and PET read-outs (SUVR, ΔSUVR). A Shapiro–Wilk test was performed to verify normal distribution of sample values (results are provided in Additional file 1). In all tests, a threshold of *p* < 0.05 was considered to be significant for rejection of the null hypothesis.

## Results

### Late-stage Anle138b treatment reduces tau deposition in hTau mice

**Fig. 1:**
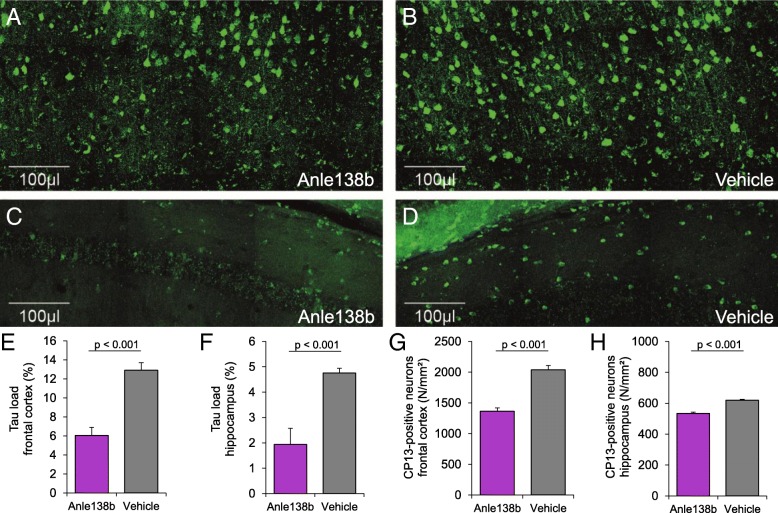
Distinct reduction of CP13-positive tau profiles by late-stage Anle138b treatment in hTau mice. Representative immunohistochemistry images of the frontal cortex (**a**, **b**) and the hippocampus (**c**, **d**) are shown for hTau mice after 3 months treatment with Anle138b or vehicle. Quantification indicated significant reductions of tau load and CP13-positive neurons in the frontal cortex (**e**, **g**) and the hippocampus (**f**, **h**). Error bars represent SEM

First, we investigated if late-stage Anle138b treatment extending over 3 months has the potential to reduce further tau accumulation in mice expressing human AD tau. One mouse of the hTau treatment group died during the baseline PET scan, and one mouse of the hTau vehicle group died between baseline and follow-up, resulting in equally sized groups of five mice for both of the treatment and vehicle groups (carrier and non-carrier). Body weight was initially 18% lower in hTau mice compared to non-carriers at baseline (*p* = 0.003), but there were no significant differences between treatment and vehicle groups at subsequent times (see Additional file 1). CP13 immunohistochemical staining revealed a clear visual reduction of tau deposition in hTau mice receiving Anle138b treatment when compared to the hTau vehicle group (Fig. 1a–d). Immunohistochemical quantitation indicated a 53% reduction of tau burden after 3 months of Anle138b treatment in the frontal cortex, relative to the burden in hTau vehicle mice (6.1% vs. 13%; *p* < 0.001, Fig. 1e). Similarly, there was 59% less tau burden in the hippocampus of the treated mice (1.9% vs 4.7; *p* < 0.001, Fig. 1f). There were fewer CP13-positive neurons in the Anle138b treatment group in the frontal cortex (− 33%; *p* < 0.001, Fig. 1g) and the hippocampus (− 14%; *p* < 0.001, Fig. 1g) when compared to the hTau vehicle group.

There was no detectable tau accumulation in non-carrier control mice, irrespective of Anle138b treatment. Table 1 gives a detailed overview of immunohistochemistry findings together with PET findings presented below.

**Table 1:** Comprehensive overview of end point immunohistochemistry and longitudinal FDG-PET findings in treated and untreated hTau mice

	hTau Anle138b	hTau Vehicle	hTau pooled Baseline	Non-carrier controls (pooled)
Immunohistochemistry Frontal cortex (%-area)	6.1 ± 1.9**	12.9 ± 1.8		0
Immunohistochemistry Frontal cortex (CP13+ neurons, N/mm^2^)	1366 ± 116**	2039 ± 153		0
Immunohistochemistry Hippocampus (%-area)	1.9 ± 1.4**	4.8 ± 0.4		0
Immunohistochemistry Hippocampus (CP13+ neurons, N/mm^2^)	534 ± 18**	619 ± 13		0
FDG-PET baseline Frontal cortex (SUVR)	0.97 ± 0.02	1.02 ± 0.09	1.00 ± 0.07^#^	1.10 ± 0.10
FDG-PET follow-up Frontal cortex (SUVR)	1.03 ± 0.05*	0.95 ± 0.03^#^		1.09 ± 0.06
FDG-PET change Frontal cortex (ΔSUVR)	+ 0.06 ± 0.06*	− 0.07 ± 0.09		+ 0.01 ± 0.05
FDG-PET baseline Hippocampus (SUVR)	0.93 ± 0.03	0.98 ± 0.10	0.96 ± 0.08^#^	1.06 ± 0.10
FDG-PET follow-up Hippocampus (SUVR)	1.00 ± 0.07	0.93 ± 0.05^##^		1.05 ± 0.07
FDG-PET change Hippocampus (ΔSUVR)	+ 0.06 ± 0.05	− 0.05 ± 0.10		+ 0.00 ± 0.05

Results summary: “*” indicates *p* < 0.05 and “**” indicates *p* < 0.005 in the direct comparison of hTau treatment and vehicle groups. “^#^” indicates *p* < 0.05 and “^##^” indicates *p* < 0.005 in the comparison of hTau groups (pooled baseline or treatment/vehicle during follow-up) versus non-carrier controls

### Metabolic decline is rescued by late-stage Anle138b treatment

**Fig. 2:**
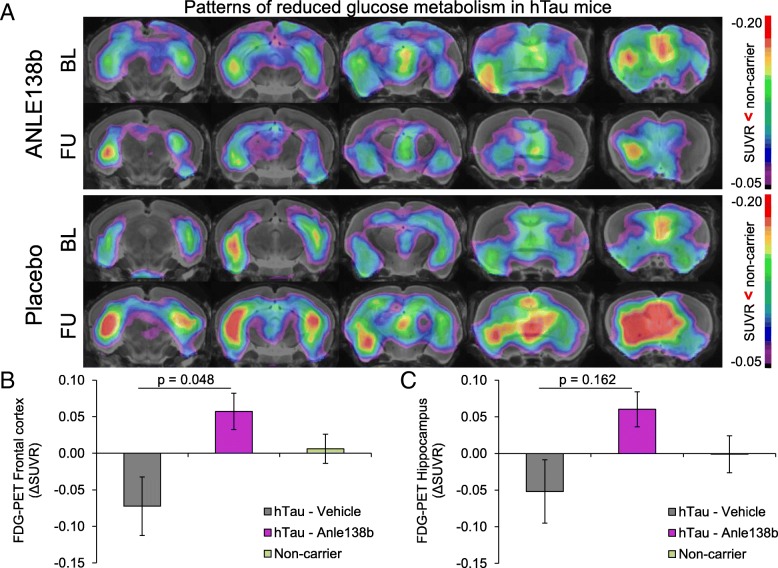
Serial FDG-PET imaging of relative cerebral metabolism (SUVR): Cerebral metabolism to FDG-PET improved at follow-up imaging 3 months after initiating late-stage Anle138b treatment in hTau mice when compared to own baseline, with an already established baseline reduction in comparison to non-carrier control mice (**a**, compare both upper rows). On the other hand, we saw ongoing metabolic decline in the vehicle-treated hTau group (**a**, compare both lower rows). Quantification of longitudinal changes in relative FDG uptake (ΔSUVR) to PET indicates normalization of cerebral metabolism with prolonged Anle138b administration, but ongoing decrease in the frontal cortex (**b**) and the hippocampus (**c**) in the hTau vehicle group. Non-carrier controls do not show any relevant changes of relative FDG uptake over time. Data are adjusted for baseline imaging. BL baseline at 14.5 months of age, FU follow-up at 17.5 months. Error bars represent SEM

Next, we aimed to elucidate if late-stage Anle138b treatment has the potential to prevent or rescue metabolic decline. Relative to non-carrier controls, the hTau mice already exhibited moderately reduced relative cerebral FDG uptake at 14.5 months of age (baseline) in the frontal cortex (SUVR − 9%, *p* = 0.025) and in the hippocampus (SUVR − 10%, *p* = 0.020) (Fig. 2). The drug intervention provoked a significant rescue of cerebral metabolism in the frontal cortex of treated hTau mice (ΔSUVR + 6%), whereas the vehicle-treated hTau mice showed further metabolic deterioration (ΔSUVR − 7%, *p* = 0.048; Table 1, Fig. 2). There was likewise a trend towards recovery of metabolism in the hippocampus of treated hTau mice (ΔSUVR + 6% vs − 5%, *p* = 0.162; Table 1). Non-carrier controls did not show any significant changes in cerebral metabolism during the observation period, nor was there any main effect of Anle138b treatment on metabolism (Table 1).

**Fig. 3:**
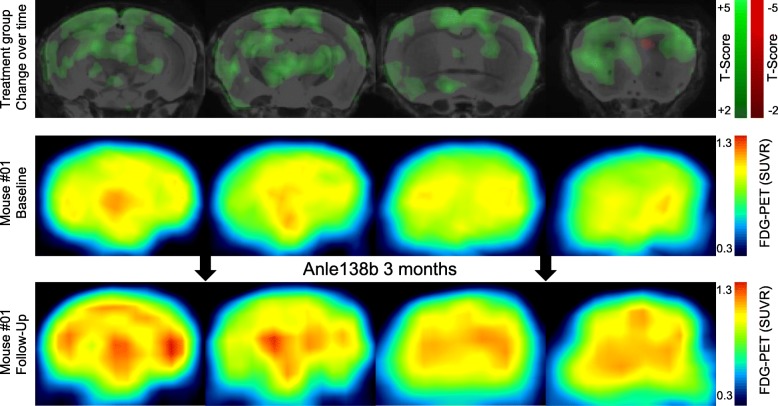
Voxel-wise analysis of metabolic rescue in the Anle138 treatment group: Direct comparison between follow-up and baseline FDG-PET imaging by statistical parametric mapping of SUVR maps (upper row) as a region-independent analysis. Green coding (coronal slices upon an MRI template) indicates voxels with a significant increase during Anle138b treatment whereas red coding indicates voxels with a significant decrease (*p* < 0.01; unc; *k* > 20). SUVR PET images of an individual example are illustrated for baseline (middle row) and follow-up (lower row) time points and show a visually discernible increasing glucose metabolism during treatment

For a region-independent statistical analysis of the potential metabolic rescue, we performed a voxel-wise longitudinal comparison between individual baseline and follow-up FDG SUVR images in the hTau Anle138b treatment group. This analysis revealed significant (*p* < 0.01, unc *k* > 20) clusters of increasing relative metabolism in the hippocampus (16,204 voxels, 4.3 mm^3^, *T*_PEAK_ 19.2), the somatomotor cortex (18,532 voxels, 4.9 mm^3^, *T*_PEAK_ 13.8), the frontal pole (15,268 voxels, 4.0 mm^3^, *T*_PEAK_ 14.2), and the thalamus (9594 voxels, 2.5 mm^3^, *T*_PEAK_ 8.9) (Fig. 3). Increasing relative cerebral metabolism upon Anle138b treatment was evident to visual inspection of SUVR images in individual hTau mice (Fig. 3).

Vehicle-treated hTau mice showed a strong loss of relative cerebral metabolism in the frontal cortex (− 13%, *p* < 0.001) and the hippocampus (− 12%, *p* = 0.007) at 17.5 months when compared to non-carrier controls. On the other hand, there was only a non-significant trend towards metabolic decline in the same contrast for the hTau Anle138b treatment group (− 6%, *p* = 0.101, hippocampus − 5%, *p* = 0.369).

### Tau pathology and metabolic decline are closely associated in hTau mice

**Fig. 4:**
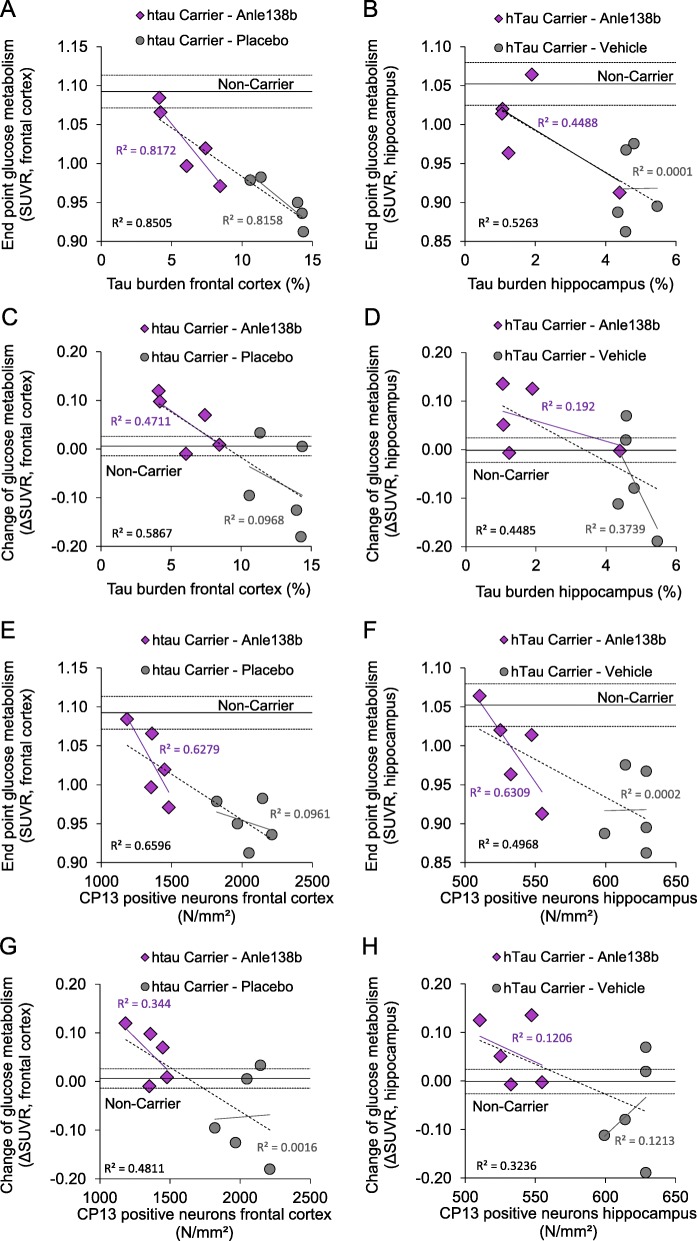
Correlation analysis of metabolic function and end point tau assessments: The end point tau burden and the number of CP13-positive neurons per unit area in the frontal cortex had strong negative correlations with end point findings (**a**, **e**) and longitudinal changes (**c**, **g**) of normalized FDG uptake in hTau mice. Corresponding plots for the hippocampus indicate the same negative correlation between tau burden and region-specific relative glucose metabolism (**b**, **d**, **f**, **h**). Purple trendlines show the linear association within the hTau treatment group, whereas the gray trendline provides the linear association for the hTau vehicle group. The dashed black line illustrates the linear association of combined hTau mice. Associated *R*^2^ values are indicated in the same color. Solid and dotted lines of non-carrier controls show mean ± SEM

Finally, we asked if our observations of attenuated metabolic decline in the treated mice are indeed attributable to reduced tau deposition. To test this, we performed a correlation analysis between read-outs of FDG-PET SUVR and tau burden in both of our target areas. Importantly, end point tau burden as assessed by immunohistochemistry in the frontal cortex had an extraordinarily strong negative correlation with the corresponding end point FDG-PET signal within the complete group of hTau mice (*R* = − 0.92, *p* < 0.001, Fig. 4a). There were also negative correlations when considering the hTau vehicle (*R* = − 0.90, *p* = 0.036) or the hTau treatment group (*R* = − 0.90, *p* = 0.035) separately, meaning that metabolic function deteriorated in concert with tau deposition. Furthermore, we also observed a negative association between the longitudinal change of relative FDG uptake and the end point tau burden (*R* = − 0.77, *p* = 0.010, Fig. 4c), which gives additional evidence for the interrelationship of the two biomarkers. End point FDG-PET (*R* = 0.81, *p* = 0.004) and longitudinal changes of relative FDG uptake (*R* = 0.69, *p* = 0.026) were also negatively correlated with the number of CP13-positive neurons in the neocortex, when considering all hTau mice. Findings in the hippocampus mirrored the results for the frontal cortex, but with lesser correlations (Fig. 4b, d, f, h).

## Discussion

We present the first study documenting an in vivo proof of rescued metabolic function to longitudinal FDG-PET in mice expressing human tau after receiving a late-stage oligomer modulator treatment. Furthermore, our end point immunohistochemical data give clear evidence that tau deposition was reduced in treated mice, in proportion to the rescue from metabolic decline. Thus, Anle138b holds promise to improve even established decline of glucose consumption by promoting reduction of CP13-positive hyperphosphorylated tau aggregates and less neurons with CP13-positive tau accumulation, which may bode well for future translational interventional studies of human AD.

Anle138b is already of proven efficacy in lowering tau deposition in the 4R mouse PS19 tauopathy model and caused an increased survival rate in these mice, when administered in chow from weaning to terminal disease [7]. The aim of the current study was to prove the drug’s performance when initiated rather as a late-stage treatment of tauopathy in an AD-like mouse model characterized by paired helical 3R/4R tau and slow disease progression resembling aspects of the human disease [14]. Late-stage initiation seems more relevant to translation into clinical testing, as patients will likely have an already established tau load at the time of diagnosis. Tau deposition in the neocortex and the hippocampus of our hTau mice was more than halved by Anle138b treatment (− 53%/− 59%) in comparison to vehicle fed mice, an effect consistent with earlier observations in similarly treated PS19 mice. Thus, we observed the anticipated beneficial effect of Anle138b oligomer modulator treatment on AD-like tau pathology also for this paradigm of late-stage treatment initiation. Nevertheless, the regional distribution of pathology and neuronal dysfunction in these hTau mice (neocortical and hippocampal) closely reflects the topology of human AD tau deposition. In contrast, PS19 tauopathy mice primarily exhibit pathology in the hindbrain, which leads to early death due to motor impairment and respiratory failure [26]. The regional pattern of tau deposition in our hTau mice also matched the pattern of baseline FDG-PET deficits, which clearly indicated reduced relative FDG uptake in the hippocampus and frontal cortex, already evident at the baseline examination at 14.5 months (Fig. 2a).

The feasibility of detection and monitoring loss of cerebral metabolism by serial FDG-PET in tau model mice was also well-supported by a recent study [13] showing decreased FDG-PET uptake in transgenic tau-VWL mice already at 11 months, which further progressed to 19 months of age. Therefore, we aimed in the present study to untangle a crucial question: does an intervention against the primary pathology caused by the transgene in the mouse model rescue or rectify the AD-like metabolic decline, where reduced FDG uptake is a surrogate for progressive failure of neuronal function? This is an important consideration in light of several failed AD vaccination trials targeting β-amyloid pathology [27, 28], which succeeded in clearing the therapy target from the brain but failed to improve cognition as their primary end point. The association between cognitive performance and neuronal dysfunction/injury is modulated by diverse factors and moderated by cognitive reserve [29]. In this regard, biomarkers of neuronal injury are important read-outs, as they resemble the desired functional therapy outcome. FDG-PET, which traces cerebral glucose consumption, is widely used for the assessment of neuronal injury and diagnosis of neurodegenerative diseases [30] and has served for therapy monitoring in AD drug trials [31]. Most diagnostic schemes of AD employed by international working groups now include biomarkers of neuronal injury together with Aβ and tau protein deposition [10–12]. Preclinical FDG-PET imaging has the clear advantage of delivering robust values even in individual mice and allows adjustment for varying baseline measures [19].

Our findings of attenuated metabolic decline with Anle138b treatment initiated at a late disease stage are compelling as they show inhibition or even dispersal of established tau aggregates, along with rebound of the previously impaired cerebral metabolism (Fig. 3). This means that the loss of brain energy metabolism arising from tau pathology is recoverable to some degree after rectifying the tauopathy. In clinical translation, sporadic AD patients will likely initiate treatment at an established disease stage, similar to our late-stage treatment paradigm as tau pathology is already established in mild cognitive impairment. Thus, the ability to halt or even improve metabolic function at late disease stages is an invaluable property for drugs targeting tau pathology, as in the present Anle138b study of tauopathy mice. This stands in contrast to preclinical results with γ-secretase-modulation and β-secretase-inhibition, which clearly lost their effectiveness once amyloid β-amyloid pathology was established [32, 33]. Nevertheless, potential differences between the mouse model and the situation in human AD need to be considered for interpretation of the results as the investigated mouse model exhibits only tau pathology, without β-amyloid accumulation. Thus, it might be more difficult to halt or improve metabolic decline by late-stage AD treatment in humans, given that the onset of tau pathology is likely triggered by initial amyloid accumulation [34].

Importantly, we find a clear negative association between the end point tau burden in the brain of hTau mice and the extent of impaired cerebral metabolism to longitudinal FDG-PET (Fig. 4). These data support a physiological linkage between AD-like tau pathology and metabolic decline. The rescue of metabolic decline after initiating oligomer treatment strengthens further this mechanistic concept. Previous human studies using PET and assays of tau in cerebrospinal fluid [3, 4] also suggested an association between tau pathology and metabolic decline, despite lacking the histopathological gold standard assessments. Thus, to our knowledge, we provide the first demonstration of a direct correlation between metabolic decline and AD tau deposition, as assessed by gold standard immunohistochemistry. Interestingly, the neocortical FDG uptake of the two hTau mice with lowest tau pathology after Anle138b treatment attained the range of non-carrier control mice (Fig. 4a). Future studies with large sample sizes might be able to define a threshold of tau pathology manifesting in neuronal dysfunction. Keeping in mind earlier investigations showing a direct linkage between metabolic changes and tau phosphorylation [35], we note as a limitation that we cannot entirely rule out a direct influence of the observed metabolic changes in FDG-PET on the rate of tau phosphorylation. Subsequently, alterations of pS202 phosphorylation could have had an impact on the intensity of the CP13 immunohistochemical staining, which was however qualitatively similar in the CP13-positive neuronal population. However, we also observed a clear treatment effect on the number of CP13-positive neurons, and we do not deem it likely that some non-specific treatment effect on global metabolic changes would completely ameliorate the CP13 signal of single neurons.

Future preclinical studies of Anle138b treatment might properly include tau PET to allow longitudinal monitoring of tau pathology in hTau mice. We previously established ^18^F-THK5117 PET in different tau mouse models [25], but were unable to see specific PET signal in untreated hTau mice even at 17.5 months of age (data not shown). This failure probably relates to the rather high detection threshold of first-generation tau PET ligands (~ 10%) and the very low magnitude of advanced forms of tau (discussed below) in hTau mice, which represent the target of arylquinoline tau ligands. Nonetheless, the current development of second-generation tau PET ligands [36] with reduced off-target binding and higher signal to noise ratio will potentially solve this technical issue. Recent establishment of the SV2A PET assay of synaptic density [37] might further enhance future study designs by affording a more direct index of synaptic integrity compared to FDG-PET. Although the FDG-PET signal is a valid surrogate for synaptic activity [38], it can also be altered by factors such as neuroinflammation or blood glucose levels. The current results call for inclusion of spatial learning tasks in future Anle138b studies with larger sample sizes.

Among the limitations of our study, we note that for technical reasons biochemical analyses of tau concentration were unavailable. Furthermore, tau pathology was only assessed by a single immunohistochemical measure targeting pS202, since Gallyas staining did not give a specific tau signal in this material (data not shown). The apparently negative Gallyas staining in untreated hTau mice at 17.5 months of age suggests only that there was an unexpectedly low degree of advanced tau aggregate forms in these mice. We speculate that our housing of the transgenic mice in a pathogen-free environment for more than 10 months before imaging might have had an impact on the severity of pathology expressed in this model.

## Conclusion

Late-stage initiation of oligomer modulation by Anle138b effectively ameliorated AD-like tau pathology in hTau mice while rescuing the declining cerebral metabolism to FDG-PET. Late-stage treatments with efficacy despite established brain pathology at the initiation of treatment are highly desirable for AD, and the present preclinical results are encouraging for the use of agents like Anle138b.

## Additional file

Additional file 1: Results of normal distribution testing and longitudinal measures of body weight. (DOCX 16 kb)

## Data Availability

The datasets used and/or analyzed during the current study are available from the corresponding author upon reasonable request.

## Abbreviations

18F: Flour-isotope with mass number 18
3R: Three repeat
4R: Four repeat
57Co: Cobalt-isotope with mass number 57
AD: Alzheimer’s disease
BL: Baseline
BST: Brainstem
FDG: Fluorodesoxyglucose
FU: Follow-up
*k*: Number of voxels
Mapt: Microtubule-associated protein tau
MRI: Magnetic resonance imaging
OSEM: Three-dimensional ordered-subset-expectation-maximum
p.i.: Post injection
PBS: Phosphate-buffered saline
PET: Positron emission tomography
*R*: Pearson coefficients of correlation
SEM: Standard error of the mean
SUV: Standardized uptake value
SUVR: Standardized uptake value ratio
Tg: Transgene
TX: Transformation
unc: Uncorrected
VOI: Volume of interest
vs: Versus
Δ%: Percentage change
μPET: Small animal positron emission tomography
