# Supplementary material for: Late-stage Anle138b treatment ameliorates tau pathology and metabolic decline in a mouse model of human Alzheimer’s disease tau
Source: Alzheimers Res Ther. 2019 Aug 1;11:67. doi: 10.1186/s13195-019-0522-z (PMC6670231; doi:10.1186/s13195-019-0522-z)
Supplement: Supplementary file 1 — Results of normal distribution testing and longitudinal measures of body weight. (DOCX 16 kb) [file 13195_2019_522_MOESM1_ESM.docx]

| **Group** | **hTau Anle138b** | **hTau Vehicle** | **Non-carrier** |
| --- | --- | --- | --- |
| FDG-PET BL frontal cortex | 0.276 | 0.160 | 0.958 |
| FDG-PET BL hippocampus | 0.731 | 0.128 | 0.638 |
| FDG-PET FU frontal cortex | 0.910 | 0.825 | 0.733 |
| FDG-PET FU hippocampus | 0.800 | 0.847 | 0.254 |
| FDG-PET Delta frontal cortex | 0.497 | 0.552 | 0.658 |
| FDG-PET Delta hippocampus | 0.339 | 0.702 | 0.150 |
| CP13 burden (%) frontal cortex | 0.083 | 0.437 |  |
| CP13 burden (%) hippocampus | 0.253 | 0.059 |  |
| CP13-pos neurons frontal cortex | 0.908 | 0.444 |  |
| CP13-pos neurons hippocampus | 0.079 | 0.899 |  |

**Table S1 –** Testing for normal distribution of all data by Shapiro-Wilk test. P-values are given for all study groups.

| **Group** | **Baseline** | **+2 weeks** | **+4 weeks** | **+7 weeks** | **+10 weeks** | **+12 weeks** | **Follow-up** |
| --- | --- | --- | --- | --- | --- | --- | --- |
| hTau pooled | 29.7 ± 3.5 | 30.6 ± 3.8 | 31.6 ± 4.4 | 33.1 ± 5.0 | 34.0 ± 5.6 | 33.9 ± 6.3 | 33.6 ± 7.0 |
| hTau Anle138b | 28.9 ± 4.6 | 29.7 ± 5.0 | 30.8 ± 5.9 | 32.1 ± 6.6 | 33.2 ± 7.2 | 33.8 ± 8.1 | 33.8 ± 8.1 |
| hTau Vehicle | 30.5 ± 1.8 | 31.6 ± 1.7 | 32.6 ± 1.9 | 34.3 ± 2.1 | 35.0 ± 3.3 | 34.1 ± 4.0 | 33.3 ± 6.4 |
| t-test Anle138b vs. vehicle | 0.4862 | 0.4397 | 0.5095 | 0.4879 | 0.6237 | 0.9452 | 0.9176 |
| Non-carrier pooled | 36.1 ± 4.9 | 38.2 ± 5.6 | 37.9 ± 5.9 | 41.3 ± 4.8 | 41.0 ± 5.6 | 40.3 ± 6.2 | 39.6 ± 4.7 |
| Non-carrier Anle138b | 37.3 ± 4.8 | 39.6 ± 5.5 | 39.8 ± 4.2 | 41.6 ± 5.1 | 40.7 ± 5.3 | 40.5 ± 5.9 | 39.0 ± 5.6 |
| Non-carrier Vehicle | 34.6 ± 5.2 | 36.5 ± 6.2 | 35.6 ± 7.5 | 40.7 ± 7.5 | 41.5 ± 7.3 | 40.1 ± 8.1 | 40.5 ± 3.6 |
| t-test Anle138b vs. vehicle | 0.4410 | 0.4496 | 0.3260 | 0.8353 | 0.8491 | 0.9388 | 0.7060 |
| t-test pooled hTau vs. pooled non-carrier | 0.0031 | 0.0031 | 0.0135 | 0.0037 | 0.0155 | 0.0428 | 0.0530 |

**Table S2 –** Longitudinal assessment of body weight. Values show mean ± SD of body weight (g) of all groups of mice investigated. PET sessions were performed at Baseline (0 weeks) and Follow-Up time points (+14 weeks). P-values derive from an unpaired student’s t-test.
